# Molecular biogeography of Europe: Pleistocene cycles and postglacial trends

**DOI:** 10.1186/1742-9994-4-11

**Published:** 2007-04-17

**Authors:** Thomas Schmitt

**Affiliations:** 1 Biogeographie, Fachbereich VI, Wissenschaftspark Trier-Petrisberg, Universität Trier, D – 54286 Trier, Germany

## Abstract

The climatic cycles with subsequent glacial and intergalcial periods have had a great impact on the distribution and evolution of species. Using genetic analytical tools considerably increased our understanding of these processes. In this review I therefore give an overview of the molecular biogeography of Europe. For means of simplification, I distinguish between three major biogeographical entities: (i) "Mediterranean" with Mediterranean differentiation and dispersal centres, (ii) "Continental" with extra-Mediterranean centres and (iii) "Alpine" and/or "Arctic" with recent alpine and/or arctic distribution patterns. These different molecular biogeographical patterns are presented using actual examples.

Many "Mediterranean" species are differentiated into three major European genetic lineages, which are due to glacial isolation in the three major Mediterranean peninsulas. Postglacial expansion in this group of species is mostly influenced by the barriers of the Pyrenees and the Alps with four resulting main patterns of postglacial range expansions. However, some cases are known with less than one genetic lineage per Mediterranean peninsula on the one hand, and others with a considerable genetic substructure within each of the Mediterranean peninsulas, Asia Minor and the Maghreb. These structures within the Mediterranean sub-centres are often rather strong and in several cases even predate the Pleistocene.

For the "Continental" species, it could be shown that the formerly supposed postglacial spread from eastern Palearctic expansion centres is mostly not applicable. Quite the contrary, most of these species apparently had extra-Mediterranean centres of survival in Europe with special importance of the perialpine regions, the Carpathian Basin and parts of the Balkan Peninsula.

In the group of "Alpine" and/or "Arctic" species, several molecular biogeographical patterns have been found, which support and improve the postulates based on distribution patterns and pollen records. Thus, genetic studies support the strong linkage between southwestern Alps and Pyrenees, northeastern Alps and Carpathians as well as southeastern Alps and the Dinaric mountain systems, hereby allowing conclusions on the glacial distribution patterns of these species. Furthermore, genetic analyses of arctic-alpine disjunct species support their broad distribution in the periglacial areas at least during the last glacial period.

The detailed understanding of the different phylogeographical structures is essential for the management of the different evolutionary significant units of species and the conservation of their entire genetic diversity. Furthermore, the distribution of genetic diversity due to biogeographical reasons helps understanding the differing regional vulnerabilities of extant populations.

## Background

Distributions of animal and plant species are extremely variable in time and space [1-9]. Thus, the strong climatic oscillations of the Pleistocene [10] repeatedly forced many species to major latitudinal and/or altitudinal range shifts [1-4]. These range changes were postulated based on chorological analysis (i.e. localisation of the core areas of distributions) [1,2,11-14], and could be traced through fossil records, e.g. from pollen [15-18], charcoal fragments [19], the elytra of beetles [20-22], remains of small mammals [23] and gastropod shells [24-26]. Many of these evidences were supported by genetic analyses [5-9]. However, the resolution of such genetic analyses often exceeds that of the classical methods, and even some cases are known in which the molecular methods unravel misinterpretations of chorological analyses and shortcomings of the other classical methods [8,27-30].

In this short review, I give a brief overview of the molecular biogeography in the western Palearctic. In the following, I distinguish between three major biogeographic groups in Europe: (i) "Mediterranean" species with Mediterranean differentiation and expansion centres, (ii) "Continental" species with extra-Mediterranean centres and (iii) "Alpin" and/or "Arctic" species, which are found in alpine and/or arctic centres of retreat today. For each of these three groups, I give a brief overview of the main ice-age distributions patterns, of the postglacial range changes and their consequences on the genetic structure.

To test biogeographical patterns, one should select model species fulfilling the following criteria: (i) the species must have a sufficiently high dispersal ability to spread rapidly into newly emerging suitable habitats ensuring that the species occupies the available space, (ii) once established, populations must be large and stable to reflect large scale structures (i.e. phylogeographic signals) and not recent local population structures, and (iii) although having a high dispersal ability, the single individual has to be mostly sedentary so that the phylogeographic patterns are not blurred as for example in migratory species. These criteria are well accomplished by many butterfly and burnet species. Furthermore, quite representative examples for the biogeographical groups mentioned above are known for this group of animals, which therefore is used as preferred model group in this review.

For the classification of the biogeographical groups, I use the definition of faunal elements sensu de Lattin [2] referring to the last dispersal centre and not to the recent distribution pattern of a taxon. The latter is commonly used in plants whereas the former is often applied in zoogeography [31]. Thus, the recent distribution of a Mediterranean element sensu de Lattin [2] can reach Scandinavia in the North, but it is considered Mediterranean, if the last refugium was in the Mediterranean Basin expanding northwards in the postglacial.

## Mediterranean species

### The three major Mediterranean peninsulas of Europe as glacial refugia

**Figure 1:**
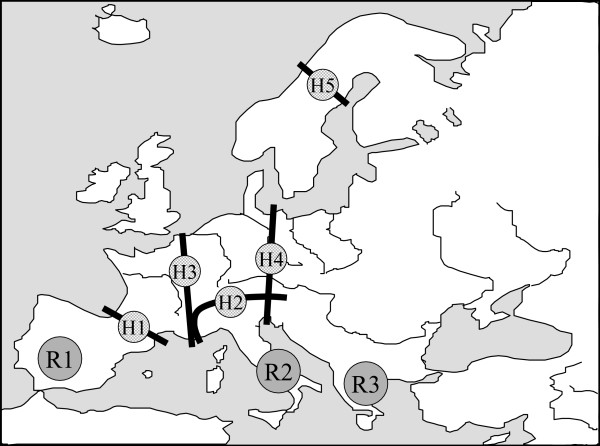
The three major Mediterranean refugial and differentiation centres of Southern Europe during the last ice-age (R1: Atlantic-Mediterranean, R2: Adriatic-Mediterranean, R3: Pontic-Mediterranean) and the geographic location of the five most important contact and hybridisation areas where different biota got into secondary contact during the postglacial range expansion processes (H1: Pyrenees, H2: Alps, H3: western Central Europe, H4: eastern Central Europe, H5: Central Scandinavia. (Based on Taberlet et al. [9] and Hewitt [5]).

**Figure 2:**
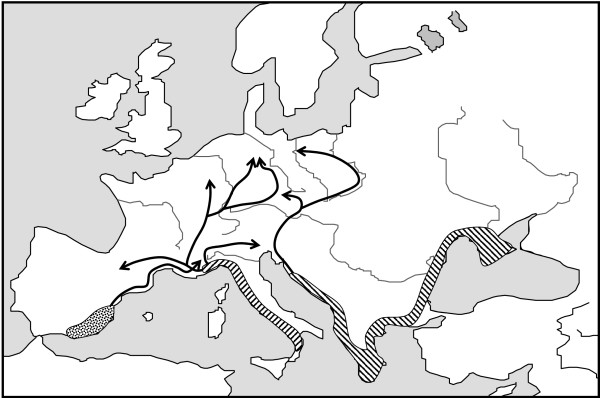
Hypothetical location of Würm ice-age refugia of the chalk-hill blues *Polyommatus coridon *(hatched area) and *P. hispana *(dotted area) in the three Mediterranean peninsulas of Southern Europe and main directions of the Postglacial range expansion (solid arrows) [32,33,122,123,177].

Almost all European species of Mediterranean origin have had their ice-age refugia in at least one of the three major European peninsulas of the Mediterranean area (i.e. Iberia, Italy and the Balkans; Figure 1), and most of the more widespread species had ice-age refugia in all the three of them [4-6,9]. In the majority of cases, the populations of these three peninsulas had no or only limited exchange of individuals and thus the populations of these disjuncted areas evolved independently often resulting in three major genetic groups, one for each of these peninsulas as shown for a large number of different animal and plant species [4,6,9]. These structures were also found in butterflies, as in the chalk-hill blues *Polyommatus coridon/hispana *complex (Figure 2) [32,33], the marbled whites *Melanargia galathea/lachesis *complex [34] and the adonis blue *Polyommatus bellargus *[35].

**Figure 3:**
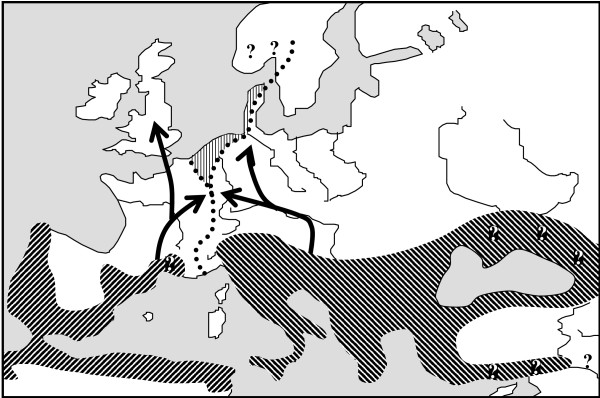
Hypothetical distribution patterns of the meadow brown *Maniola jurtina *in Europe during the last glacial maximum (dark hatched areas). Postglacial expansions are indicated by solid arrows. The postulated actual hybrid zone is shown by the hatched area. Question marks indicate lack of information concerning ancient distribution patterns in the South and present distribution of hybrid populations in the North. Redrawn from Schmitt et. al. [36]; based on Thomson [37] and Schmitt et al. [36].

However, some cases became recently known in which no genetic differentiation among these three refugial centres were detected. Thus, the meadow brown *Maniola jurtina *only shows two major genetic lineages, one Atlantic-Mediterranean lineage and a Central-Eastern-Mediterranean one (Figure 3) [36]. These allozyme data are supported by morphological data of the male genitalia and of the wings of both sexes [37]. The eastern phylogenetic group most probably is due to considerable gene flow between the Adriatic- and the Pontic-Mediterranean centres during the last ice-age, whereas the Atlantic-Mediterranean area remained isolated. In an allozyme study of the common blue *Polyommatus icarus *over major parts of Europe no differentiation among the three major European refugial areas has been detected [38], thus supporting constant gene flow for this species on a European scale even during the last ice-age. As the common blue is recently distributed as far north as northern Scandinavia [39] and the arctic parts of Asia [40], a relatively wide European distribution even during glacial periods is feasible, thus making *P. icarus *not a typical Mediterranean species.

### Refugia within refugia – genetic differentiations within the Mediterranean sub-centres

A sub-division of the large Mediterranean refugia, based on chorological analyses, was previously postulated e.g. by Reinig [41]. These assumptions have recently been supported by genetic analyses, and a large number of taxa show a strong sub-structuring within the Mediterranean sub-centres such as the Maghreb, Iberia, peninsular Italy, the Balkans and Asia Minor.

### The Atlantic-Mediterranean element

The Atlantic-Mediterranean refugial area embraces Iberia and the major part of the Maghreb [2] and thus is divided into an African and a European part by the Strait of Gibraltar, and several examples of strongly differentiated taxa north and south of this sea barrier are known, especially in amphibians as in the genera *Pleurodeles, Discoglossus, Alytes, Pelobates, Rana *and *Salamandra *[42-51]. This sea water barrier has permanently existed since the end of the Messinian Salinity Crisis about 5.33 Ma ago [52-55], and thus has strongly impeded the migration of sea water intolerant organisms like amphibians. Nevertheless, strongly differentiated genetic lineages on both sides of the Strait of Gibraltar are also known e.g. in the lizard *Psammodromus algirus *[56] and the butterfly sibling species complex *M. galathea/lachesis *[34].

In groups of organisms not being strongly restricted in their dispersal by sea (e.g. reptiles, butterflies) relatively recent (e.g. late Pleistocene) exchange of individuals is known, as in several snakes [57,58], turtles [59,60], tortoises [61], chameleons [62], butterflies [36], bark beetles [63], but also in the frog *Hyla meridionalis *[64]. The more southern and thus warmer Maghreb more often represented the centre of origin [47,57-62,64-70] than Iberia [56].

In many cases, the Maghreb populations show remarkable east-west differentiations as in the lizard *P. algirus *[56], the snake *Malpolon monspessulanus *[58] and the shrew *Crocidura russula *[70] most probably representing the frequent existence of eastern and western sub-centres of differentiation. In the turtle *Mauremys leprosa*, the two Maghreb lineages are separated by the Atlas Mts. in Morocco and Algeria with the southern lineage expanding from southern Morocco to the Mediterranean Sea in Tunisia [60]. This distribution matches the Mauretanic-Mediterranean sub-centre postulated by de Lattin [2].

In Iberia, a strong genetic differentiation into a western and an eastern group is also known for a larger number of species as in the case of *Pleurodeles *newts [47,48,66], *Discoglossus *frogs [50], the burnet moth *Aglaope infausta *[71], the lizards *P. algirus *[56] and perhaps *Acanthodactylus erythrurus *[72]. Paleoclimatology shows that the climatically most favourable areas of Iberia were in disjunct areas in the southwestern and the southeastern parts of the peninsula [73]. Therefore, it is most likely that xero-thermophilic species had two disjunct areas of glacial survival and differentiation in the Iberian Peninsula [71]. However, several of these and other taxa evolved an even more complex genetic structure beyond this simple east-west split [46,50,51,65,74-80].

Thus, the Iberian lizard species *Lacerta schreiberi *consists of four major genetic lineages, two coastal clades and two inland clades, most probably representing four disjunct areas of glacial survival in Iberia with only the northern coastal clade becoming expansive during the postglacial [74]. *Chioglossa lusitanica*, an endemic salamander species of northwestern Iberia, has a none-expansive clade in central Portugal and an expansive clade in northern Portugal and northwestern Spain [81,82]. The western lineage of *P. algirus *is further divided into a southern and a northern sub-cluster [56]. As much as six lineages are distinguished in the western Iberian endemic newt *Lissotriton boscai*, and three of these lineages are confined to southwestern Portugal [80]. These structures strongly support the existence of several sub-refugia in Iberia along the Pleistocene often resulting in remarkable differentiations between genetic lineages.

The southwest of Portugal revealed to be of special importance as refugial area. This is further supported by the rather fine-grained genetic lineages of the cyprinid *Squalius aradensis *in the Algarve (southern Portugal) [78], and the extremely high haplotype diversity of *M. leprosa *in southern Portugal [60] underlining the long-term persistence of taxa in this part of Iberia. If postglacial northwards expansions occurred in one of these lineages within Iberia, remarkable genetic impoverishment is the frequently observed result [60,63,74,81,82] as in other species on the continental scale (see below) [4].

The fire salamander *Salamandra salamandra *is also present in three lineages in Iberia, one endemic lineage in the south, another in central and northern Iberia, which is the predominant lineage of Europe, and a third one in a limited area in Cantabria that is identical with the one in the southernmost tip of Italy. The origin of the latter is still ambiguous, and the widespread lineage might have entered Iberia only in the postglacial [45]. The *Timarchia goettingensis *complex shows a highly complex genetic structure in Iberia implying a series of vicariance events and differentiation centres for this beetle [75,76].

### The Adriatic-Mediterranean element

The Adriatic-Mediterranean refugial area is represented by peninsular Italy [2]. As the geographical diversity of this area and the total land mass is considerably less in peninsular Italy than in Iberia, the phylogeographical patterns are considerably simpler in the Adriatic- than in the Atlantic-Mediterranean region. Truly Mediterranean species like the bark beetle *Tomicus destruens *[63] and the Apennine yellow-bellied toad *Bombina pachypus *[83] only survived in relatively restricted areas of Calabria and Sicily, and only expanded to more northern regions of Italy when the climate warmed in the postglacial; hereby, they lost genetic diversity. Calabria is particularly rich in strongly differentiated lineages, e.g. four in the Italian wall lizard *Podarcis sicula *[84], two in *S. salamandra *[45] and two in *B. pachypus *[83]. This underlines the great importance of this area as refugial region and its particular substructure. However, Sicily is not differentiated from Calabria in most cases reflecting the close geographic proximity with the peculiar exception of the hedgehog *Erinaceus europaeus *showing a remarkable genetic differentiation in Sicily [85].

Apart from differentiation centres in southern Italy, several species also show distinct lineages in the northern part of peninsular Italy, such as the lizard *P. sicula *[84], the grasshopper *Chorthippus parallelus *[86], the honey bee *Apis mellifera *[87], and the asp viper *Vipera aspis *[88], thus underlining the existence of additional allopatric differentiation centres in this region. In some cases different lineages expanding in Italy from the south and the north (the latter sometimes with their roots outside of Italy) have built up hybrid zones in Italy as in the case of the honey bee [87] and the bark beetle *T. destruens *[63].

### The Pontic-Mediterranean element

The Pontic-Mediterranean refugial region sensu de Lattin [2] embraces the Balkan Peninsula, Asia Minor and the east coast of the Mediterranean as far south as Palestine. For this area even Reinig [41] suggested a number of sub-centres, a hypothesis which is strongly supported by recent genetic analyses in this region. Thus, often deep genetic splits between the Balkan Peninsula and Asia Minor support the long lasting biogeographic isolation of these two areas as e.g. detected in the hedgehog *Erinaceus concolor *[85,89], the grasshopper *C. parallelus *[86], the bark beetle *T. destruens *[63] and most probably the badger *Meles meles *[90].

Several genetic data sets support Reinig's idea [41] of several sub-centres in the Balkan Peninsula. Thus, phylogeographic work on the pond turtle *Emys orbicularis *[59], the fire-bellied toad *Bombina variegata *[91], the hedgehog *E. concolor *[85,89] and the slug *Arion fuscus *[30] all support an east-west split of the Balkan Peninsula with western Illyrian and eastern Aegean phylogroups. A similar situation was found in the marbled white *M. galathea *[92]. The allozyme analysis of individuals from southeastern Europe support at least three centres of survival along the Mediterranean coasts of the Balkans: the Illyrian Coast, the Aegean Coast and the southwestern coast of the Black Sea. Whether these three centres of dispersal were linked to each other during the Würm ice-age or not is still unknown.

Old splits in the southern Balkan Peninsula even date back to the formation of the mid-Aegean trench (12 to 9 Ma ago) [93]. This event has been reported to have acted as a major factor determining the biogeographical patterns of scorpions [94,95] and reptiles [96,97]. In the scorpion *Iurus dufoureius *this vicariance is mirrored in a western group from the Peloponnisos to Crete island and an eastern clade from Karpathos island to southwestern Turkey [94]. In other species, more complex genetic patterns evolved with localised endemic lineages especially in the long-term isolated islands of the southern Kyklades and Crete as in the scorpion *Mesobuthus gibbosus *[95], *Podarcis *lizards [96,97] and land snails [98,99].

### Postglacial range expansions: the four paradigms

The postglacial re-colonisation of Central and Northern Europe by Mediterranean species followed in general four paradigm patterns [5,6,34]: (i) the hedgehog (postglacial expansion from all three southern European differentiation centres) [100], (ii) the bear (expansion of the western and the eastern lineage, but trapping of the Adriatic-Mediterranean lineage by the Alps, *nota bene *that the expansion of the eastern lineage in the bear example is not from the Pontic-Mediterranean region but from an eastern continental differentiation centre) [101], (iii) the butterfly (expansion of the Adriatic- and the Pontic-Mediterranean lineages, but trapping of the Atlantic-Mediterranean lineage by the Pyrenees) [34] and (iv) the grasshopper (major expansion to Central Europe only from the Balkans and trapping of the Atlantic- and Adriatic-Mediterranean lineages by the Pyrenees and Alps, respectively) [86]. 

These paradigms are frequently repeated in many animal and plant species, thus the "hedgehog" paradigm e.g. in oaks *Quercus ssp*. [102], the silver fir *Abies alba *[103] and the house mouse *Mus musculus *[104], the "bear" in e.g. the shrews *Sorex araneus *[105,106] and *Crocidura suaveolens *[9] and the water vole *Arvicola terrestris *[9] and the "grasshopper" e.g. in the black alder *Alnus glutinosa *[107], the common beech *Fagus sylvatica *[108] and the newt *Triturus cristatus *[109]. Only three examples are actually known for the "butterfly" paradigm: the marbled whites *M. galathea/lachesis *complex [34], the chalk-hill blues *P. coridon/hispana *complex (Figure 2) [33] and, based on a small data set, the adonis blue *P. bellargus *[35]. The latter paradigm might be a common phylogeographical structure for quickly expanding species, so that the Italian lineage might have expanded rapidly, passing westwards south of the Alps and afterwards expanding all over southern France, so that this space had been occupied when individuals of the Iberian lineage started to pass the Pyrenees. However, further examples are needed for this paradigm's support.

### Genetic consequences of Postglacial range expansion

The Postglacial range changes had major impact on the genetic conditions of the affected species. Therefore, it is of major importance for the genetic texture whether a species is expanding phalanx like, stepping stone or leptokurtic [110].

In the first case, the species perform only weak genetic differentiation during the process of range expansion, often without loss of genetic diversity. Such structures are characteristic for species with wide ecological amplitudes and were also found in common butterfly species like in the green-veined white *Pieris napi *[111,112], the common blue *P. icarus *[38], the small tortoiseshell *Aglais urticae *[113], the meadow brown *M. jurtina *[36] and the marbled white *M. galathea *[34]. All these species do not show significant losses of their genetic diversity from their expansion centres towards their recent northern distribution border.

On the contrary, stepping stone and leptokurtic dispersal are responsible for the evolution of genetic patterns along the process of expansion often going along with the loss of genetic information during the processes of expansion, as shown for many animal and plant species [114-121]. Such a genetic structure is characteristic for species with specific habitat requirements so that no phalanx can expand, but only some few individuals reach so far unoccupied emerging habitats, as also known for the chalk-hill blue *P. coridon*. This species shows a linear loss of genetic diversity from the south to the north in its Pontic-Mediterranean lineage [122] and a stepwise loss of genetic diversity in the Adriatic-Mediterranean lineage (i.e. no loss of genetic diversity over larger distances as from southern France to northeastern France, and abrupt reduction of genetic diversity as a decrease of 10% of the number of alleles resulting from the passing through of the Burgundian Gate between the Vosges and the Jura Mts.) [123].

### Contact zones in Europe

As a consequence of postglacial range expansion in the group of species of Mediterranean origin, a network of contact zones is spread over Europe (Figure 1). However, these contact zones between different genetic lineages are not randomly scattered over the continent, but show a systematic pattern. Thus, the mountain systems of the Pyrenees and Alps, often acting as dispersal barriers, represent two important areas where different biota met, but also three other regions of Europe show a high number of secondary contact zones between genetic lineages [5,6,9]: (i) western Central Europe along the French-German border region and along the western Alps (Figure 3), (ii) eastern Central Europe along the eastern borders of Germany and through the eastern Alps and (iii) running east-west through central Scandinavia.

For butterflies, the meadow brown *M. jurtina *has a typical contact zone in western Central Europe where the Atlantic-Mediterranean lineage meet with the Central-Eastern-Mediterranean group (Figure 3), which is supported by two allozyme studies, the morphology of the male genitalia and three wing patterns [36,37]. Along the French-German border regions, no natural obstacles are separating these two lineages; apparently, these lineages have met in that area and the populations, once set up by one evolutionary lineage, remained mostly stable over time; however, some hybridisation in Belgium and the Netherlands is possible, based on the morphology of the male genitalia [37].

Evidence for the eastern Central European contact zone exists e.g. for two butterfly species. The two major genetic lineages of the chalk-hill blue *P. coridon*, based on allozyme analysis and wing patterns, meet in this region (Figure 2) [32,33]. Mostly all along this contact zone the natural conditions for the survival of this warm-loving species of calcareous grasslands [124,125] limit its existence: an acid sandy area in northeastern Germany, acid cold mountains ranges along the Czech-German border region and the watersheds of the eastern Alps [32] so that this contact zone might partly be shaped by natural expansion obstacles too, but not so extreme as in the cases of the Alps and Pyrenees. The Czech-German border mountains also act as contact zone for the woodland ringlet *Erebia medusa*; these mountains apparently were reached by an eastern and a western lineage early in the postglacial (more details on the molecular biogeography of this continental species in the next chapter), but the high elevations of these mountains could only be colonised by the species some time after its arrival so that the main ridges mostly represent the contact zone between both lineages [126].

## Continental species

Formerly, a considerable proportion of the European fauna was considered of eastern Palearctic origin with postglacial expansion from Siberian or Mandshurian refugia throughout Asia to Europe [2]. This postulate has been questioned for a long time e.g. by Varga [3] and recent publications give rising evidence even for the existence of cryptic northern refugia north of the Alps and Pyrenees [127]. Thus, genetic analyses unravel extra-Mediterranean ice-age refugia in Europe in a series of other animal species as in the adder *Vipera berus *in the Carpathian Basin, areas adjoining the Alps and one or more areas in central Europe [128], in the frog *Rana arvalis *in the Carpathian Basin [129,130], the fish *Cottus gobio *in Germany [131], the voles *Microtus arvalis *in the Black Forest region and maybe elsewhere in Central Europe [132], *Microtus agrestis *[133] and *Clethrionomys glareolus *[28], the ant *Formica pratensis *in the Pyrenean region [134], the slug *Arion fuscus *in the region of the High Tatras [30] and the nematode *Heligmosomoides polygyrus *[29]. Thus, extra-Mediterranean ice-age refugia in Europe might be a much more common feature in temperate continental species than previously thought, but more investigation in the field of continental species is urgently needed [127].

A particularly interesting and well studied species in the group of continental species represents the woodland ringlet *E. medusa*. There is good evidence based on allozyme data that this species had multiple Würm ice-age differentiation centres around the glaciated Alps, in the Carpathian region and in the Balkan Peninsula ([27,135]; TS unpublished data). This glacial distribution pattern in *E. medusa *and most probably in other similar species too (one further good example with strikingly similar genetic pattern being the adder [128]) might be due to the particular climatic conditions in Europe during the ice-ages: (i) the climate was predominantly much drier than today and (ii) the continentality of the climate was increasing westwards [10]. Therefore, not only the low temperatures, but also lack of precipitation restricted the distribution of many animal and plant species during the glacial phases of the Pleistocene. While the temperature might have been the major restriction for the distribution of the above discussed Mediterranean species, the dryness might have been even more important for many of the taxa out of the group of continental species. *Erebia medusa *[136] and *V. berus *[137] for example are recently distributed in Central Asia in regions with extremely cold winters so that the temperatures in Central Europe during the ice-ages might not have been the main constraint for their distributions. In contrast to temperature, the dryness of Central Europe might have been the limiting factor for the species' glacial distributions in Europe. Therefore, the survival of the woodland ringlet as well as several other species [30,131,132,134,138,139] in relatively small areas adjoining the water-donating high mountain systems like the Alps and Carpathians is feasible as well as in the proximity of the high mountain systems of the Balkan Peninsula ([27,135]; TS unpublished data).

However, there is evidence for some species having colonised Europe postglacially from eastern dispersal centres. This is mostly the case for typical Taiga species like the Russian flying squirrel *Pteromys volans *[140] and the ant *Formica lugubris *[134], in which postglacial expansion from eastern Palearctic Würm ice-age refugia is probably based on their genetic texture, but not in a species like the Siberian jay *Perisoreus infaustus*, in which western and eastern Palearctic populations belong to strongly differentiated phylogroups [141]. In butterflies, the scarce heath *Coenonympha hero *shows a gradient of declining genetic diversity from the southern Ural Mts. over Estonia to southern Sweden, but no split into different genetic lineages [142]. This structure supports a glacial centre of survival for this species in the southern Urals and postglacial range expansion westwards to Sweden.

The adder *V. berus *shows in its mtDNA haplotypes the peculiar structure of highly differentiated genetic lineages in Europe, but one lineage stretching from Finland and the Baltic countries throughout Russia to the Sakhalin island [128]. This strongly supports a centre of origin in the western Palearctic and relatively recent (maybe even postglacial) expansion throughout Asia, thus showing an opposed pattern to the classical Siberian element definition sensu de Lattin [2].

## Arctic and/or Alpine species

In the past it was thought that "Arctic" and/or "Alpine" species in many cases show an almost opposed pattern to Mediterranean species: they expand during cold stages and become restricted to mountain or Nordic refugia during interglacials [1,2,12]. Recent work on arctic and alpine species has revealed that this pattern was an oversimplification. In fact, there is evidence that some arctic animal and plant species survived the last glaciation even at high latitudes or even north of the extensive ice shields; the evidence is strong for Beringia and northeastern Asia, weaker for the High Nearctic and doubtful for northern Europe [8,143,144]. In general, two largely different patterns are found in this biogeographical group:

(i) Some species were widely distributed throughout the glacial steppes between the northern ice shield and the glaciers in the mountains of the South, so that a large scale gene flow was only interrupted by the postglacial disjunction into different high mountain systems and the arctic (i.e. arctic-alpine disjunct species). This classical glacial distribution pattern of arctic-alpine species is well supported by fossil evidence [145], but the genetic evidence is still relatively poor [146].

Thus, a common origin of *Dryas octopetala *from the southern European mountains, Iceland, Scotland and Scandinavia is demonstrated by AFLP analysis [147]. Close genetic relation between arctic populations and such from the southern European mountains are also known for *Saxifraga oppositifolia *[143], *Minuartia biflora *[148] and the lycosid *Pardosa saltuaria *group [149]. Postglacial immigration into Scandinavia from source populations in the eastern Alps was invoked for *Ranunculus glacialis *because of genetically depauperate populations relative to the otherwise quite similar alpine ones [150]. The genetic structure of the boreo-montane bog fritillary *Proclossiana eunomia *[151] implies postglacial colonization of Fennoscandia from an eastern refugium, maybe representing the Siberian differentiation centre *sensu *de Lattin [2]. An eastern origin of Scandinavian populations is also likely in *Trollius europaeus *[152] and *D. octopetala *in the northern part [147]. Some genetic evidence for a wide distribution in the glacial steppes and semi-deserts is available for the burnet moth *Zygaena exulans *with a rather weak differentiation between Alps and Pyrenees [153] and the water beetle *Hydroporus glabriusculus *with no significant genetic differences among populations from Britain and Scandinavia [154]. The species surviving in these environments have to be adapted not only to rather cold, but also to extremely dry steppic conditions with the latter maybe excluding a large number of the typical alpine species from the periglacial steppe belt.

(ii) Other species have probably been subject to disjunct distribution patterns during glacial and – in many cases – interglacial phases, thus accumulating much larger differentiation than species of the first group [146,149,150,152,155-168]. Especially the dryness of the glacial steppes and not the low temperatures might have excluded a large number of such cold adapted species from these areas. Therefore, many of these species became restricted to the wetter areas in the proximity of the glaciated mountain systems [146,167,168]. Thus, multiple (often four) refugia were scattered for various species (e.g. *R. glacialis, Phyteuma globulariifolium, Androsace alpina, Pritzelago alpina, Drusus discolor, Erebia epiphron*) along the margins of the southern and eastern Alps [157,164,166-168].

Remarkable differentiation of the inner-alpine populations of *Eritrichum nanum *[155,160], *Saponaria pumila *[161], *Rumex nivalis *[165], *Erinus alpinus *[159], *S. oppositifolia *[169] and *Androsace wulfeniana *[164] even give genetic support for in situ survival in the Alps (i.e. nunatak survival).

Many of the smaller European high mountain systems own their endemic genetic lineages. The populations of these mountain areas often simply performed altitudinal shifts respective to climate changes and no major long distance colonisation. Thus their differentiation centres likely existed at the foot hills of these mountains throughout the last glaciation and often even much longer, as shown for the Cantabrian Mts. (*P. eunomia *[151], *Pritzelago alpina *[163]), the Pyrenees (*P. alpina *[163], *D. discolor *[167], *Pardosa oreophila *[149], *E. epiphron *[168]), the Tatra Mts. (*D. discolor *[167]), the Stara Planina (*P. eunomia *[151]) and Pirin & Vitosha (*Pardosa drenskii *[149]).

**Figure 4:**
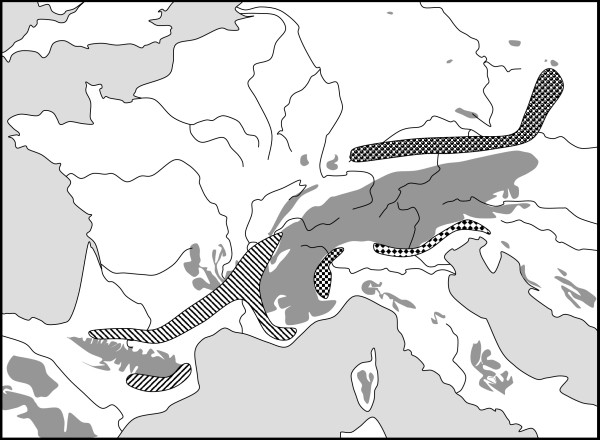
Hypothetical distribution patterns of the mountain ringlet *Erebia epiphron *in western Europe during the last glacial maximum. Grey areas show mountain areas above 1000 m asl. Hatched areas: Western major group, dotted areas: eastern major group. Redrawn from Schmitt et al. [168].

However, there is good genetic evidence for glacial links between the Pyrenees and the southwestern Alps (*Anthyllis montana *[156]*, Erebia cassioides *[170]*, E. epiphron *[168]), the northern Alps and the Jesenik Mts. (*E. epiphron *[168]), the northern Carpathians and the northeastern Alps (*P. alpina *[163]*, Ranunculus pygmaeus *[148]) and the mountains of the western Balkan Peninsula and the southeastern Alps (*A. montana *[156]) during glacial periods. The postulated five centres of differentiation of the mountain ringlet *E. epiphron *illustrating the complexity of the patterns found in this biogeographical group are presented in Figure 4[168].

## Conservation implications

There is increasing evidence that the population genetic constitution is highly important for the individual fitness [171-173]. Meta-analyses have shown that the sensitivity of the populations to extinction is negatively correlated with the respective regional genetic diversity resulting from their biogeographic history [174]. Therefore, continental species in general are more endangered in the western part of Europe than in the East, whereas Mediterranean species perform better in the genetically richer South than in the North [174].

These analyses underline the high importance for conservation of the populations existing in the areas of the Quaternary refugia: these populations as a whole own most of the total genetic diversity and thus the evolutionary potential of a species [175,176]. The frequently observed genetic differentiations of marginal races at the high-latitude leading edge are therefore of considerably less evolutionary importance than the low-latitude rear edge populations because (i) the high-latitude edge lineages are mostly due to recent (i.e. postglacial) adaptation processes and not long-term evolution [176] and (ii) most of these lineages may simply disappear leaving no descendants when the climate will become cooler again [175]. However, the high diversity of different biogeographical patterns point out that each species has reacted independently to Pleistocene climatic changes in terms of persistence in a refugium, migration rates and colonisation routes [175]. Therefore, generalisations for conservation measures should be made with extreme care.

## Conclusion

Europe shows a great variety of different biogeographical patterns with (i) "Mediterranean", (ii) "Continental" and (iii) "Arctic/Alpine" being the predominant ones.

Within the "Mediterranean" group, (i) one or (ii) more (sometimes phylogeographically highly complex) genetic lineage(s) evolve during glacial isolation within a single Mediterranean peninsula or (iii) two or more peninsulas share a single lineage. Postglacial range expansions follow four main patterns: (i) "Hedgehog", (ii) "Bear", (iii) "Butterfly" and (iv) "Grasshopper" depending on whether Alps and/or Pyrenees acted as postglacial expansion barrier or not. As a consequence of range expansions, biota predominantly got into contact in five regions of Europe: (i) along the Pyrenees' main ridge, (ii) along the Alps' main ridges, (iii) in western Central Europe running north-south, (iv) in eastern Central Europe running north-south and (v) in central Scandinavia running east-west.

In the group of "Continental" species, two major biogeographical types may be distinguished: (i) species with non-Mediterranean glacial refugial and differentiation centres in Europe, with special importance of the continental areas of the Balkan Peninsula, the Carpathian Basin and the perialpine areas around the Alps, with many of these centres of distribution being geographically rather restricted, but in general numerous, and, to a lesser extent, (ii) postglacial expansion from eastern differentiation centres located in Asia with the western most possibility being the southern Ural Mts. The latter species group is mostly composed of typical Taiga species.

The third important pattern, the group of "Arctic" and/or "Alpine" species, consists of two rather different subgroups: (i) species that have been widely distributed in the periglacial steppes during the glacial periods and (ii) taxa that also had disjunct distribution patterns during the ice-ages. The former group splits into (i) species only retreating northwards in the Postglacial (i.e. arctic species) and (ii) species retreating northwards and into the mountain systems in the south (i.e. arctic-alpine species *s*. *str*.). The latter group, showing disjunct ice-age distribution patterns, is mostly absent from the high latitudes and often shows a variety of genetic lineages stemming from different glacial differentiation centres, but now coexisting within a single high mountain system, as best demonstrated for the Alps. Furthermore, several biogeographic links between mountain systems are known in this group best explained by glacial distribution patterns as in the links between (i) Pyrenees and southwestern Alps, (ii) Carpathians and northeastern Alps as well as (iii) the Dinaric Mountain system and the southeastern Alps.

Apparently, we know quite a lot about the molecular biogeography of Europe, but many aspects still remain enigmatic. Therefore, further investigation in this field is urgently needed to really understand and conserve the highly complex biogeographical constitution of the European continent.
